# Optimization of Natural Antioxidants Extraction from Pineapple Peel and Their Stabilization by Spray Drying

**DOI:** 10.3390/foods10061255

**Published:** 2021-06-01

**Authors:** Sofia C. Lourenço, Débora A. Campos, Ricardo Gómez-García, Manuela Pintado, M. Conceição Oliveira, Diana I. Santos, Luiz C. Corrêa-Filho, Margarida Moldão-Martins, Vítor D. Alves

**Affiliations:** 1 LEAF, Linking Landscape, Environment, Agriculture and Food, Instituto Superior de Agronomia, Universidade de Lisboa, Tapada da Ajuda, 1349-017 Lisbon, Portugal; sofiaclourenco@isa.ulisboa.pt (S.C.L.); dianaisasantos@isa.ulisboa.pt (D.I.S.); lucaalbernaz@gmail.com (L.C.C.-F.); mmoldao@isa.ulisboa.pt (M.M.-M.); 2 CBQF—Centro de Biotecnologia e Química Fina—Laboratório Associado, Escola Superior de Biotecnologia, Universidade Católica Portuguesa, Rua Diogo Botelho 1327, 4169-005 Porto, Portugal; deborancampos@gmail.com (D.A.C.); rgarcia@porto.ucp.pt (R.G.-G.); mpintado@porto.ucp.pt (M.P.); 3 Centro de Química Estrutural, Instituto Superior Técnico, Universidade de Lisboa, Av. Rovisco Pais, 1049-001 Lisboa, Portugal; conceicao.oliveira@tecnico.ulisboa.pt

**Keywords:** solid-liquid extraction, phenolic compounds, pineapple peel, encapsulation, maltodextrin, spray drying

## Abstract

Pineapple peel still contains an important amount of phenolic compounds and vitamins with valuable antioxidant activity. In this way, the aim of this study was the recovery of the bioactive compounds from pineapple peel using environmentally friendly and low-cost techniques, envisaging their application in food products. From the solid-liquid extraction conditions tested, the one delivering an extract with higher total phenolic content and antioxidant capacity was a single extraction step with a solvent-pineapple peel ratio of 1:1 (*w*/*w*) for 25 min at ambient temperature, using ethanol-water (80–20%) as a solvent. The resulting extract revealed a total phenolic content value of 11.10 ± 0.01 mg gallic acid equivalent (GAE)/g dry extract, antioxidant activity of 91.79 ± 1.98 µmol Trolox/g dry extract by the DPPH method, and 174.50 ± 9.98 µmol Trolox/g dry extract by the FRAP method. The antioxidant rich extract was subjected to stabilization by the spray drying process at 150 °C of inlet air temperature using maltodextrin (5% *w*/*w*) as an encapsulating agent. The results showed that the antioxidant capacity of the encapsulated compounds was maintained after encapsulation. The loaded microparticles obtained, which consist of a bioactive powder, present a great potential to be incorporated in food products or to produce bioactive packaging systems.

## 1. Introduction

The amount of byproducts derived from fruit processing represents socio-economic and environmental problems with low significant industrial and commercial value. Due to the low protein content, seasonally restricted availability, and high perishability of some fruits, these byproducts can even be poor for animal feed. For these reasons, many strategies have been suggested for the reduction, reuse, and recycle of industrial fruit waste [1].

Pineapple (*Ananas cosmosus* L., family Bromeliaceae) is a fruit that grows in the tropics and sub-tropics and is consumed in many parts of the world as fresh fruit, juice, canned, jelly, and dried product. According to Roda et al. [2], during industrial processing of pineapple, about 75% of the original fruit is converted in byproducts, where the peel is the largest proportion generated and is not usually utilized for commercial purpose.

Previous reports in the literature demonstrated that the peel of some fruits, which remain after processing, contain a higher quantity of antioxidants when compared with the edible portion [3,4,5]. Pineapple peel remains with a high nutritive value, including a considerable amount in sugars (sucrose, glucose, and fructose), organic acids (malic, citric, and quinic acids), essential minerals (K, Mg, Ca), cellulose, hemicellulose, pectin, lignin, enzymes, and antioxidants, namely flavonoids, vitamin A and C [6,7,8,9]. A few number of researchers have already been exploring several applications for this byproduct, namely functional ingredients based on its nutritional and medicinal properties, as a source of proteolytic enzymes or for cellulose and bioethanol production [10,11,12]. However, studies regarding the applications of this byproduct in food products are limited [6]. Based on the properties revealed, pineapple peel bioactive compounds are supposed to be promising ingredients in what concerns their potential applications as preservatives, substitutes for synthetic antioxidants, and used in the development of functional foods [13,14,15].

The extraction of bioactive compounds is the most important step for converting them into useful products. The literature has several reports of peel extracts obtained from conventional techniques such as solvent-liquid extraction, such as those from citrus peel [16], banana peel [17], pomegranate peel [18], passion fruit [19], and grape skin [20]. A number of promising techniques are also used in the extraction of bioactive compounds from fruits peel such as ultrasound-assisted extraction (UAE) [5,21], microwave-assisted extraction (MAE) [20], pressurized liquid extraction (PLE) [22], supercritical fluid extraction (SFE) [23], etc. [13]. Some of these non-conventional techniques include the less use of hazardous solvents, high extraction yield, and fewer hours to achieve satisfactory recoveries. However, these are generally difficult and more expensive techniques to apply.

Although studies dealing with the extraction of phenolic compounds from pineapple peel have been performed [24], the present work was focused on using this source on industrial food application, which requires the use of extraction methods that allow the maximum extraction yield using efficient, nontoxic, environmentally friendly, and low-cost techniques [25,26]. In addition, the effectiveness of these compounds depends on their stability in food processing and storage. Some studies using pineapple fruit extract for direct application to enzymatic browning inhibition of fresh-cut products as a coating agent have been conducted [27,28,29,30]. However, in the industry, generally it is not directly applied after the extraction, and stabilization processes between the production and the use of these compounds is required. In this sense, the aim of this study is to optimize the extraction conditions of the bioactive compounds from the pineapple peel, based on a direct and economical approach, and to explore the possibility of stabilizing this valuable source of natural antioxidants for further application in food products.

## 2. Materials and Methods

### 2.1. Raw Material

Fresh pineapple peel of Sweet Gold variety from fresh-cut processing was provided by Campotec IN, Torres Vedras, Portugal. These byproducts were transported at a temperature of 4 °C to the laboratory, where they were stored in bags under a vacuum at −80 °C.

### 2.2. Extraction Method

Defrosted pineapple peel was minced, and simple solid-liquid extraction processes were applied using water or ethanol-water mixtures as solvents in dark conditions. After contact, the mixtures were subsequently filtered through a muslin cloth and the permeate was centrifuged at 7000 rpm for 10 min at 15 °C (HERMLE Labortechnik 383 K, Wehingen, Germany). The clear supernatant (liquid extract) was collected and stored in amber flasks, avoiding light, and then flushed with nitrogen, until further analysis or encapsulation.

#### 2.2.1. Aqueous Extraction: Experimental Design and Statistical Analysis

To assess the most favorable experimental conditions for the extraction of hydrosoluble bioactive compounds from the pineapple peel, the response surface methodology (RSM) was applied. A central composite rotatable design (CCRD), with the solvent:pineapple peel ratio (S:S) (1:1–1:6) and the extraction time (t) (5–45 min) as independent variables was performed. The total phenolic content (TPC) was the response variable. A total of 12 experiments were performed randomly in order to avoid systematic errors (Table 1). The central point was carried out in quadruplicate to assess the experimental error [31,32,33].

The experimental data was fitted to a second-order polynomial Equation (1) to predict the dependent variable and total phenolic compounds content (TPC), through a stepwise multiple regression analysis using the StatisticaTM v. 8.0 software (StatSoft Inc., Tulsa, OK, USA, 2007).

TPC = b_0_ + b_1_t + b_11_t^2^ + b_2_S:S + b_22_S:S^2^ + b_12_tS:S (1)

where b_0_ is the interception, b_1_ and b_2_ are the linear regression coefficients, b_11_ and b_22_ are the quadratic coefficients, and b_12_ is the interaction coefficient. A *p*-value < 0.05 was considered significant. The three-dimensional response surfaces as a function of independent variables, t and S:S, were obtained.

#### 2.2.2. Aqueous Extraction with Optimized Conditions

After optimizing the conditions for the extraction of hydrosoluble compounds, the effect of other parameters was studied, using the solvent:pineapple peel ratio (1:1 *w*/*w*) for 25 min: (i) The effect of temperature—ambient temperature (T_amb_) and 100 °C (T_100_); (ii) UAE at ambient temperature; and (iii) Consecutive extraction steps at ambient temperature. UAE was performed on an ultrasonic water bath (Elma Transsonic Analog T700/H, Singen, Germany) at a working amplitude of 35 Hz. Consecutive solid-liquid extraction steps were performed at ambient temperature, by adding fresh minced peel to the supernatant recovery in the previous step. Three extraction steps were caried out.

#### 2.2.3. Influence of Solvent Type

The influence of solvent type was investigated using the solvent:pineapple peel ratio (1:1 *w*/*w*) during 25 min with (i) water and (ii) ethanol-water mixtures (E:W 10:90 and E:W 80:20 *w*/*w*) at ambient temperature (T_amb_), 100 °C (T_100_) and 50 °C (T_50_).

The extraction processes were performed according to Section 2.2.2. The ethanol of the hydroalcoholic extracts after centrifugation was evaporated under low pressure at 40 °C (Rotavapor R II—Buchi, Germany), to obtain a liquid aqueous extract.

### 2.3. Stabilization of the Bioactive Extracts by Encapsulation Using Drying Processes

After selecting the best extraction methods, the respective extracts were stabilized by three ways: (i) Direct addition of wall material to the aqueous extracts followed by spray drying; (ii) Extracts freeze drying; and (iii) Encapsulation of freeze dried powders by spray drying.

#### 2.3.1. Direct Addition of Wall Material to Extracts Followed by Spray Drying

Maltodextrin 4–7 DE (5 wt%) was dissolved in the selected extracts (one produced using water and the other using an ethanol-water mixture (E:W 80:20 *w*/*w*) as solvents, at ambient temperature, during 25 min, with a solvent:pineapple peel ratio of 1:1. The wall material dissolution was carried out under stirring during 15 min at 8000 rpm (ARE, Velp). The solutions were fed to a spray dryer model SD-05 (Lab-plant SD-05, Huddersfield, England, 1995), at co-current flow regime, equipped with a 0.5 mm diameter nozzle, and a drying chamber with 500 mm in height and 215 mm in diameter. The feed flow rate was set at 3.7 mL/min and the inlet air temperature was 150 °C, selected according to the range of values referred in the literature [34,35,36,37]. The dried particles were stored in sealed flasks in the dark at 5 °C until further analysis.

#### 2.3.2. Freeze Drying

One portion of the extract obtained using water as a solvent, at ambient temperature for 25 min, and with a solvent:pineapple peel ratio of 1:1 *w*/*w*, was freeze dried in aluminum sample pans using a laboratory freeze dryer Telstar Lyo Quest under a 0.1 mbar pressure over a period of 48 h. The freeze dried powder obtained was stored away from light after nitrogen flushing.

#### 2.3.3. Encapsulation of Freeze Drying Powder by Spray Drying

After freeze drying, a feed solution with a volume of 25 mL was prepared by dissolving the freeze dried powder in distilled water (2754.56 mg GAE freeze dried extract/L solution) and maltodextrin (5 wt% on a freeze dried extract basis). The mixture was left under constant stirring 8000 rpm for 15 min at ambient temperature, in a magnetic stirrer (ARE, Velp). The mixture was spray dried using the same conditions as previously described in Section 2.3.1. The particles were stored away from light until analyzed.

### 2.4. Spray Drying and Microparticles’ Characterization

#### 2.4.1. Morphological Characterization of Microparticles

The morphology of the particles was evaluated according to the method described by Lourenço et al. [38]. The microparticles were coated with gold/palladium and analyzed using a Hitachi S2400 scanning electron microscope (Hitachi High-Tech, Fukuoka, Japan) operated at 15 kV.

#### 2.4.2. Preparation of Extracts from Microparticles

For the preparation of the extracts from microparticles, the method described by Rocha et al. [39] was applied with some adjustments. The microparticles (200 mg) were added to 10 mL of water and stirred with an ultraturrax homogenizer (IKA Labortechnik, T25 Basic, Staufen, Germany) at 13,500 rpm during 3 min to disrupt the particles. The suspension was kept away from light at 10 °C for 12 h. Afterwards, the solutions were centrifuged (7000 rpm, 15 min, 4 °C) and the supernatant was stored in amber flasks.

#### 2.4.3. Particles Loading

The extracts obtained in the previous step were filtered with a 0.2 µm syringe filter and the absorption of the filtrate was measured at 280 nm (UNICAM, UV/Vis Spectrometer–UV4, Alva, UK). Using a previously elaborated standard curve with gallic acid, the total phenolic content (TPC) of the particles was calculated, and the loading was expressed as the mass of GAE per mass of particles.

### 2.5. Analytical Methods

#### 2.5.1. Total Soluble Solids, pH, Color, and Moisture Content

The optimized extracts obtained were characterized in relation to their physicochemical properties. Total soluble solids (TSS) were determined with a table digital refractometer (Atago PAL-1, Tokyo, Japan) and expressed as °Brix. The pH value was measured at ambient temperature using a pH meter (CRISON Basic 20, Barcelona, Spain). For the color evaluation, aluminum crucibles were filled with the extract and the color was measured directly using a Minolta ChromaMeter (Model CR-400, Osaka, Japan) by measuring L*, a*, b* color space (CIELAB) parameters, as described by Nadzirah et al. [40]. The moisture determination was performed by drying in an oven (Binder, New York, United States) at 115 °C to constant mass.

#### 2.5.2. Total Phenolic Content (TPC)

The TPC was determined using two different methods. It was determined by direct measurement of the absorbance at 280 nm (UNICAM, UV/Vis Spectrometer–UV4, Alva, UK) [41]. This is a fast and reproducible method for total phenolics evaluation [42]. The Folin-Ciocalteau method modified by Mokrani et al. [43], was also used. Briefly, 0.02 mL of the different extracts were mixed with 1.68 mL of Folin-Ciocalteau’s reagent, diluted in water. After 5 min, 0.3 mL of sodium carbonate solution was added, and the tubes were immediately vortexed. The reaction was kept for 2 h in the dark and the absorbance measured at 765 nm, against an ethanol 95% blank (UNICAM, UV/Vis Spectrometer–UV4). The TPC was estimated using a calibration curve with gallic acid as a reference, at a concentration ranging between 0 to 0.5 mg per millimeter.

The total phenolic content was determined using the standard curves elaborated for the two methods and expressed as gallic acid equivalent (mg GAE/L or GAE/g dry weight).

#### 2.5.3. Identification of Phenolic Compounds

##### Identification by HPLC

The HPLC analysis of phenolic compounds from the extracts, and of those encapsulated by spray drying, was performed on a Waters e2695 separations module system interfaced with the Photodiode array UV/Vis detector (PDA 190–600 nm), according to the method described by [44]. The HPLC analysis was performed using a reversed-phase C18 column coupled with a guard column (pore size 100 Å, particles size 5 µm, lengths 4.6 × 150 mm) containing the same stationary phase (Symmetry^®^ C18, Waters, Mildford, MA, USA). Chromatographic separation of phenolic compounds was carried out using the mobile phase A consisting of water-methanol (Panreac, Barcelona, Spain)-formic acid (Merck, Darmstadt, Germany) (92.5:5:2.5% *v*/*v*/*v*) and mobile phase B consisting of methanol-water-formic acid (92.5:5:2.5% *v*/*v*/*v*). A gradient program was used as follows: Gradient elution started at 100% mobile phase A and ended at 55% after 50 min, between 50 and 55 min the mobile phase A returned to 100% and remained at this percentage for 4 min (until 59 min). The compounds were analyzed at a continuous flow rate of 0.5 mL/min and the injection volume was 20 µL. Detection was achieved using a diode array detector (Waters, Mildford, MA, USA) with spectral data from all peaks accumulating in the 200–600 nm range in 2 nm intervals. The chromatograms were recorded at 280, 320, and 360 nm. Three injections were performed for each sample. Compounds identification was performed by comparison of retention time and optical absorbance of commercial standards.

##### Mass Spectrometry

The ethanol-water mixture (80:20) extract was also analyzed by HPLC-DAD-MS on an Ultimate 3000 SD with a diode array detector coupled online to a LCQFleet ion trap mass spectrometer with an ESI source (Thermo Scientific, San Jose, CA, USA), and on an Ultimate 3000 RSLCnano system (Thermo Scientific, San Jose, CA, USA) interfaced with a QqTOF Impact II mass spectrometer equipped with an ESI source (Bruker Daltonics, Bremen, Germany). Chromatographic separation was performed on a C18 reversed-phase Symmetry column (150 × 4.6 mm, 5 μm particle size; Waters, Massachusetts, United States), at a constant temperature of 30 °C. The mobile phase consisted of 0.1% formic acid (*v*/*v*) in water (A) and in methanol (B). The elution gradient (A:B) was as follows: 93.5:6.5 from 0 to 2.5 min; 55:45 at 50 min; 0:100 from 55 to 58 min; and 93.5:6.5 from 59 to 65 min. A flow rate of 500 µL/min was used, and the LC eluent was introduced into the ESI source in a post-column splitting ratio of 3:1. The UV absorbance was monitored at 280 and 350 nm. MS data were acquired in the ESI negative mode in a mass range between 100–1000 Da. For details about LC-DAD-MS and UHPLC-MS/MS settings, see Sanchez et al. [45] and Sharif et al. [46], respectively.

#### 2.5.4. Antioxidant Activity

##### DPPH Assay

The antioxidant activity was quantified using the 1,1–diphenyl-2-picrylhydrazyl (DPPH) radical, as described by Lourenço et al. [38]. Briefly, a volume of 0.1 mL of the extracts was added to 3.9 mL of the working DPPH solution and the absorbance was measured at 515 nm after 40 min. The activity was expressed as the Trolox equivalent antioxidant capacity (TEAC), quantified in this study as µmol of Trolox equivalents per mass of dry particles or as µmol Trolox/mg GAE. In the case of the pineapple peel extract before encapsulation, it was expressed as per mass of dry extract.

##### FRAP Assay

The evaluation by the FRAP method was carried out according to the methodology used previously by Lourenço et al. [38]. Briefly, a volume of extract (90 µL) was mixed with 270 µL of deionized water and with 2.7 mL of the working FRAP solution. After the reaction in a water bath at 37 °C for 30 min, the absorbance was measured at 595 nm. The activity was expressed as the Trolox equivalent antioxidant capacity (TEAC) and on an iron sulfate basis.

### 2.6. Statistical Analysis

The results are expressed as the means ± standard deviation of triplicate assays. One-way analysis of variance (ANOVA) followed by Tukey’s multiple range tests were applied to determine statistical differences among samples, using the StatisticaTM v. 8.0 software (StatSoft Inc., 2007, Tulsa, OK, USA). Significant levels were defined with α = 0.05.

## 3. Results

### 3.1. Efficiency of Extraction Methods

#### 3.1.1. Aqueous Extraction

The experimental results of response variable (TPC) are shown in Table 2. The regression analysis was performed in order to statistically evaluate the quadratic models applied. Table 2 shows the linear and quadratic effects of the independent variables, and their interaction, on responses for the studied dependent variable. Determination coefficients and significance of lack of fit are also presented.

**Figure 1:**
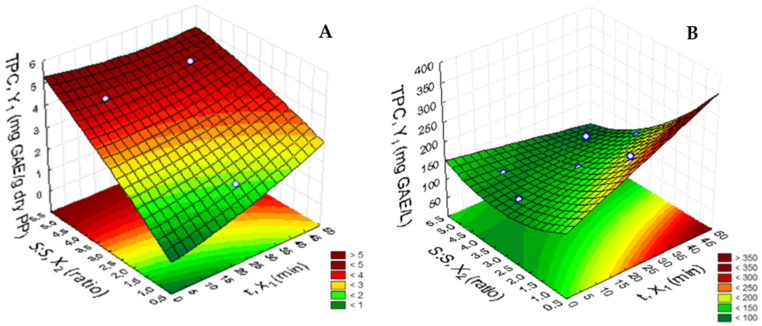
Response surfaces for the effect of time and water:raw material ratio on the total phenolic content (TPC) of extracts expressed as mg GAE/g dry pineapple peel (**A**) and mg GAE/L (**B**). Abbreviations: PP—pineapple peel; S:S—solvent:pineapple peel ratio; t—time.

The quadratic model used for TPC in the pineapple peel aqueous extracts was successfully fitted to the experimental data within a confidence level of 95%, with high values for R^2^ and R^2^_adj_. TPC was shown to be mainly dependent on the linear solvent:pineapple peel ratio effect (*p* < 0.05) and marginally dependent on the linear time effect (*p* < 0.1) (Table 2). According to Table 1, TPC varied from 1.71 to 4.66 mg GAE/g dry pineapple peel. The response surface plot illustrated in Figure 1A shows that the combination which produced the highest recovery of phenolic compounds from peel is that with a high water:raw material ratio (6:1 *w*/*w*).

Concerning the influence of the water:raw material ratio, Pinelo et al. [47] and Pompeu et al. [48] have shown that the total amount of solids obtained in the liquid increased with the higher solvent-raw material ratio, despite the solvent used. These authors state that these results are consistent with mass transfer principles, where the mass transfer rate is improved when a higher solvent-raw material ratio was used, due to an increased diffusion rate before the equilibrium is reached.

Although in the results obtained there is a higher phenolic content with respect to the raw material weight on the dry basis, a more dilute extract was produced. Thus, to choose the conditions that enable the highest concentration of target compounds, the focus was changed to the response of TPC with respect to the extract volume (mg GAE/L) (Figure 1B). In this case, TPC ranged from 101.98 to 257.85 mg GAE/L (Table 1). The maximum concentration was observed with a solvent:pineapple peel ratio of 1:1 *w*/*w* (Table 2). Table 2 summarizes the results of the analysis of variance and shows that the linear water:raw material and time effects are statistically significant (*p* < 0.05). The calculated model was able to explain 89% of the results in the case of TPC (mg GAE/L).

The time of extraction is an important parameter to be optimized even in order to minimize the energy cost of the process and to decrease the processing time [49,50]. Although the maximum concentration of TPC was observed at 50 min, according to Belwal et al. [51] and Wissam et al. [52], prolonged extraction times could compromise the decomposition of phenolic compounds. Therefore, it was assumed that the time of 25 min was a good compromise in terms of phenolics extraction and its possible degradation. Based on the results obtained, the conditions of solvent:pineapple peel ratio of 1:1 and 25 min were selected for the subsequent extraction studies.

#### 3.1.2. Effect of Type of Solvent, Temperature, and Extraction Process

Several studies reported the effect of other parameters, such as solvent type, method of extraction and temperature, as well as on the extraction efficiency of bioactive compounds [53,54,55,56,57]. Particularly, the phenolic compounds present in fruit byproducts are highly reactive. In this way, extraction conditions could modify the phenolic profile of the extract [47,49,50,54].

**Figure 2:**
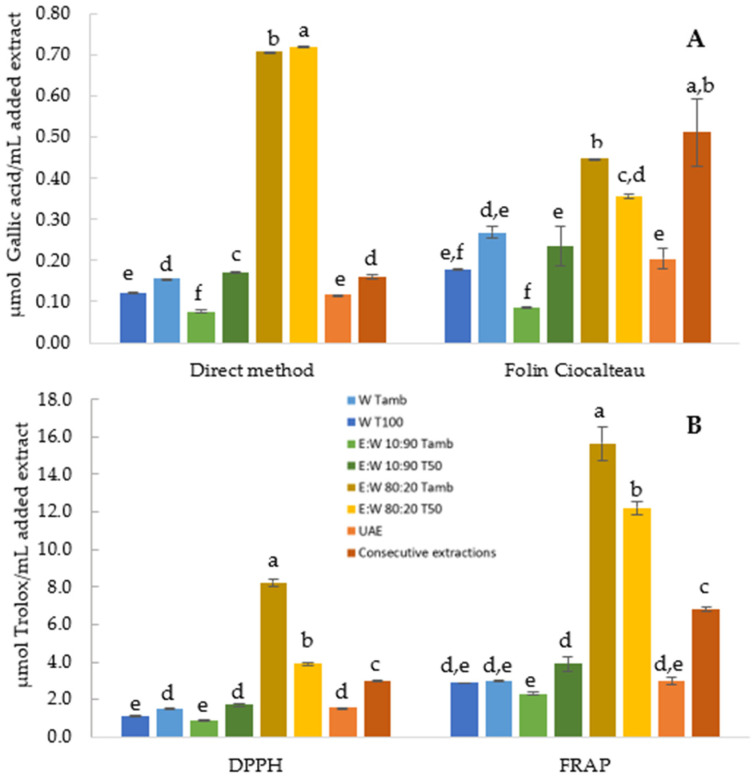
Total phenolic content (**A**) and antioxidant activity (**B**) of different pineapple peel extracts produced by the diverse methods studied. Abbreviations: W—water as solvent; E:W—ethanol:water mixture as solvent; Tamb, T100 and T50—extraction at ambient temperature, 100 and 50 °C, respectively; UAE—ultrasounds assisted extraction. Means with different letters indicate significant differences in extracts (*p* < 0.05).

Although water is the greenest solvent for the extraction of pineapple peel, it has limitations in the amount of phenolic compounds extracted. Thus, using the solvent:pineapple peel ratio and time selected in the first studies, the effect of other parameters, namely the type of solvent, temperature, and extraction methods were investigated. The TPC evaluated by direct and Folin-Ciocalteau methods, and antioxidant activity (AOA), measured by DPPH and FRAP methods, of the different extracts obtained, are shown in Figure 2.

Clearly, there is not one standard solvent for performing the solid-liquid extraction of natural antioxidants from fruit sources. In the literature, solvents such as water, ethanol, methanol, acetone, and ethyl acetate or a mixture of solvents for the extraction of the bioactive compounds from plant matrices have been reported. The choice depends on the nature of the compound to extract, with the effectiveness influenced by the solubility in a particular solvent used [24,58,59]. According to some studies, it is expected that the use of water as a solvent, presents lower phenolic extraction values [24]. However, this study intends the use of non-organic solvents allowing a lower cost extraction, using a more direct and greener technology for large scale industrial production. Moreover, the use of organic solvents limits the application in the food industry, mainly due to the food safety issues [60]. Still, among solvents, ethanol is known as a good solvent for polyphenol extraction. This compound was categorized as generally recognized as safe (GRAS), which allows its application in food products [5]. Therefore, in the present study, pineapple peel extracts were obtained using distilled water 100% (W) and ethanol:water (E:W) mixtures at different ratios—E:W 10:90 and E:W 80:20 (*w*/*w*).

The effect of different solvents on the recovery of TPC at ambient temperature is shown in Figure 2. In general, all the three solvents could extract phenolic compounds. The results revealed that the application of these solvents resulted in extracts with significant differences (*p* < 0.05) between them regarding TPC. The use of E:W 10:90 rather than water, did not increase substantially the AOA and TPC of the extracts. However, among the solvents tested, E:W 80:20 extracted the highest amount of phenolic compounds. According to Do et al. [61], the polarity of the solvent plays a key role in increasing phenolics solubility. Ethanol is a lower polar molecule than water that enhances the solubility of low polarity solutes. Then, the use of water in combination with ethanol allowed a formation of a medium that ensures a better extraction [24]. The results of TPC suggest that most of the phenolics presented in pineapple peel have moderate polar characteristics [62]. Further, increasing the ethanol concentration to 80% caused a significant (*p* < 0.05) increase in AOA. Other authors confirmed these results, showing in their works that 80% ethanol was the optimal concentration for phenolics extraction [63].

According to Figure 2, the extraction temperature demonstrated a significant (*p* < 0.05) effect on the phenolic content measured by the direct method and on the AOA evaluated by DPPH. As depicted, a significant (*p* < 0.05) increase of the AOA was perceived with water and E:W 10:90, with increasing temperature. The rise of the temperature provides the increase of the solubility, a decrease of the viscosity of the solvent and surface tension, increasing the mass transfer rate of the compounds to be extracted [24,52,64]. In contrast, the TPC did not change substantially when increasing the temperature, for E:W 80:20 extracts. In addition, the AOA of these extracts exhibited a decrease with the increasing extraction temperature. In this case, the highest temperature used was 50 °C for safety reasons. Although higher temperatures may improve the recovery with some solvents, increasing above a certain limit may degrade the phenolic compounds and newly formed compounds that absorb at the same wavelength may appear. Additionally, it may also promote solvent loss by vaporization, which decreases the yield and therefore increases the cost of extraction [65]. Thus, it would be more convenient to use ambient temperatures for extraction of bioactive compounds from pineapple peel.

Among the factors affecting the efficiency of the extraction, the number of extraction steps should be considered. Several authors have studied sequential extraction processes and concluded that consecutive extraction steps may be more effective in terms of phenolic content yield [66]. According to Benmeziane et al. [59], three consecutive extractions from fresh table grape appeared suitable, with percentages of 60, 22, and 13%, respectively. Additionally, one more stage only extracts a small amount of these compounds. A similar result was obtained by Garmus et al. [66], with the highest yields for the phenolics extraction from pepper-rosmarin leaves in three sequential steps compared to the yields obtained in the one-step extraction. The use of at least two consecutive extractions, even with the combination of aqueous-organic solvents with different polarities was also recommended by Pérez-Jiménez et al. [67].

However, in the works referred, a fresh solvent was added in each extraction step in order to maintain the same raw material. In this work, to obtain an extract as much concentrated as possible in phenolic compounds, fresh raw material was used in each step, to which the supernatant of the previous step was added. After three consecutive extraction steps, the results showed that the AOA and TPC of the extract obtained were lower when compared to that of extracts produced with 80% ethanol:water mixture (Figure 2).

The interest has been shifted in the past years towards the use of non-conventional extraction techniques [68]. In this study, the ultrasound assisted extraction technique was used to compare the performance of conventional solvent extraction (with 100% water), since it is the most chosen non-conventional extraction technique in the literature for fruits [69,70]. The ultrasound is a potential method to enhance the extraction yields by facilitating the penetration of the solvent into the solid matrix, and can be completed within minutes [64,71]. In a study conducted by Pan et al. [72], the impact of ultrasound assisted extraction on antioxidant recovery from pomegranate peel compared with conventional extraction was evaluated. The ultrasound significantly improved the antioxidant yield by 24% and reduced the extraction time by 90%. In fact, according to the literature, the use of this technique can reduce energy consumption and extraction time and increase the yield to 6–35% higher than that obtained using traditional extraction techniques [5,64]. However, contradictory results were observed in this work. The results of the ultrasound assisted extraction (Figure 2) exhibited a phenolic content and AOA similar (α = 0.05) to the extraction with water at ambient temperature and heating.

From environmental and ease of operation point of views, water should be used as a solvent for the extraction of bioactive compounds from pineapple peel. However, extraction with ethanol-water (80:20 *w*/*w*), followed by ethanol evaporation under a vacuum, appears to be the most effective extraction method.

#### 3.1.3. Major Physicochemical Properties of Selected Extracts

Table 3 lists the major physicochemical properties of the selected extracts produced previously, water extract (W) and hydroalcoholic extract with 80% ethanol (E:W 80:20), produced at ambient temperature.

Both extracts contain large amounts of water (95.5 and 93.7%) and an acidic pH (Table 3), similar to the pH found on pineapple (peel and pomace) reported by Selani et al. [73] using the same pineapple variety (pH = 3.86).

The results of color are also identical with the results reported by Nadzirah et al. [40] regarding physicochemical properties of different pineapple peel maturity stages. These authors reported that the peel color has a linear relationship with total soluble solids (TSS), correlated with total sugars as well as acids, by the increasing trend of these variables with the storage period of pineapple. An increase in the “b*” and “L*” values was observed in E:W 80:20 compared to the W extract, while a decrease was observed in the “a*” values. These results indicate that the color of E:W 80:20 extract becomes more yellowish and with lower brightness than the W extract.

Concerning the TPC values, in terms of Folin results, the extracts showed TPC values of 4 and 7 mg GAE/g dry extract or using another base, 21 and 32 mg GAE/100 g fresh peel, for W and E:W 80:20 extracts, respectively. These results were similar to those reported in the literature (7.98 mg GAE/g dry weight pineapple peel extract) using hexane and methanol for the pineapple peel extraction with different pineapple varieties [74], and using the mixture of the residues (pulp, seed, and pulp) with methanolic extraction 9.1 ± 1.3 mg GAE/g dry weight [6].

This byproduct contains many antioxidants, which makes it difficult to evaluate each component separately. Therefore, several methods have been developed to measure the total AOA, resulting in different results depending on the method used. However, since each method gives different results, is difficult to compare with the data reported in the literature. Moreover, as TPC, different AOA values are frequently observed among the same method and sample due to the cultivars, ripeness, heterogeneity of the sample, processing, as well as the extraction conditions and techniques of each study [75]. More than half of the total antioxidant capacity in fruits comes from phenolic compounds that reveal scavenging efficiency on free radicals and reactive oxygen species [76]. According to the research by Li et al. [74], gallic acid was found to be the most active DPPH radical scavenger and the ferulic acid the least active, both present in pineapple peel. In this study, with the DPPH method, the AOA was 7.28 and 91.79 µmol Trolox/g dry extract in W and E:W 80:20 extracts, respectively. Martínez et al. [77] showed lower values of DPPH for pineapple peel using a different solvent (4.8 µmol Trolox/g of dry extract). Martínez et al. [77] reported that the AOA by the FRAP method pineapple peel extracted with different solvents (methanol, ethanol, and acetone) was 6.2 µmol Trolox/g of dry extract, less than that obtained in this study.

The AOA and TPC of the extracts envisage several potential applications. However, the high moisture content and the total sugar content, increases the probability of deterioration by microorganisms, in addition to the reaction with oxygen or other deteriorative reactions over time. For this reason, preservation and stabilization methods are necessary, not only to maintain the activity of compounds over time, but also to increase the ease of handling, transport, and storage before application in food products.

### 3.2. Effect of Drying Stabilization Processes

#### 3.2.1. TPC and AOA

Despite its beneficial content, the effectiveness of the natural bioactive compounds present in pineapple peel extracts depends on its stability during storage and food processing [78]. The application of bioactive compounds in food is very limited due to their fast degradation. For this reason, drying methods such as freeze and spray drying are techniques recommended to preserve these sensitive compounds. Table 4 and Table 5 present the impact of these two thermal processes on TPC and AOA, respectively, of W (water) and E:W 80:20 extracts.

Spray drying is a well-known and commonly used technique to dry sensitive materials into a powder form [79]. Particles produced with the direct addition of wall material to the water extract (W) did not show any AOA of the encapsulated bioactive when compared to the initial extract (data not shown). This fact may be related to the low content in phenolics of the initial extract [80]. To overcome this problem, the water extract was freeze dried (FD-W), resuspended in water, followed by the addition of wall material (maltodextrin), and afterwards processed by spray drying to obtain SD-W/FD particles.

Drying may result in some structural and chemical changes that can affect quality properties such as AOA. As can be observed, regarding the AOA, the phenolic compounds present in FD-W and in particles obtained by SD-W/FD exhibited less antioxidant capacity than that of initial extract, measured by the FRAP assay. There was approximately 60% of AOA loss by the FRAP method in both processes compared to the initial extract. Different results were obtained using the DPPH assay. According to this method, the phenolic compounds present in solid FD-W and in SD-W/FD particles have similar AOA to the compounds of the water extract (W) before drying. Different AOA valuation methods are expected to produce diverse results since they assess the scavenging capacity of different molecules. While DPPH is a synthetic radical used to evaluate the ability of the compounds for scavenging free radicals, the FRAP assay is based on the ferric ion reducing activity of antioxidant compounds.

Comparing the AOA of solid FD-W and of SD-W/FD particles, no differences were observed between them. Based on the observations, the use of combined freeze and spray drying may have potential, as the AOA is maintained, and the extract is delivered encapsulated in microparticles. This fact enhances its stability over time and facilitates its use in a higher scale. For application in the food industry, it is advantageous to have the dry solid in a powder format, and spray drying is the best technique to adjust its properties, such as the size of the particles. For this reason, the E:W 80:20 extract was stabilized directly by spray drying.

As shown in Table 5, the particles obtained by the spray drying process containing the ethanolic extract E:W 80:20 (SD-E:W 80:20) present a higher AOA compared to that obtained with the water extract after being freeze dried (SD-W/FD), and there was no loss of antioxidant capacity after encapsulation. In fact, there is a slightly higher AOA observed with the DPPH method after the encapsulation, which is probably due to the underestimation of the loading of the particles, as AOA is expressed per mass of TPC (mg GAE).

In this study, the results do not show an evident correlation between TPC and AOA. According to the literature, some studies also report no correlation between these two parameters in plant extracts [81,82]. This low relationship may be caused by the different effectiveness of different phenolic compounds as antioxidants [82]. For example, flavonoids, one of the phenolic compounds classes referred to be present in pineapple peel, have more hydroxyl groups compared to ferulic acid, and therefore have better electron donating and AOA [76,83].

In summary, two stabilized extracts with interesting AOA and good potential for further studies were identified: SD-E:W 80:20 and SD-W/FD. Their stabilization was carried out by spray drying, after the direct addition of maltodextrin (wall material) to the liquid extract before drying, ending with a bioactive powder composed of microparticles. From these, the encapsulated SD-E:W 80:20 extract presents more advantages, as its particles show a higher AOA and are obtained with only one drying step.

#### 3.2.2. Particles Morphology

**Figure 3:**
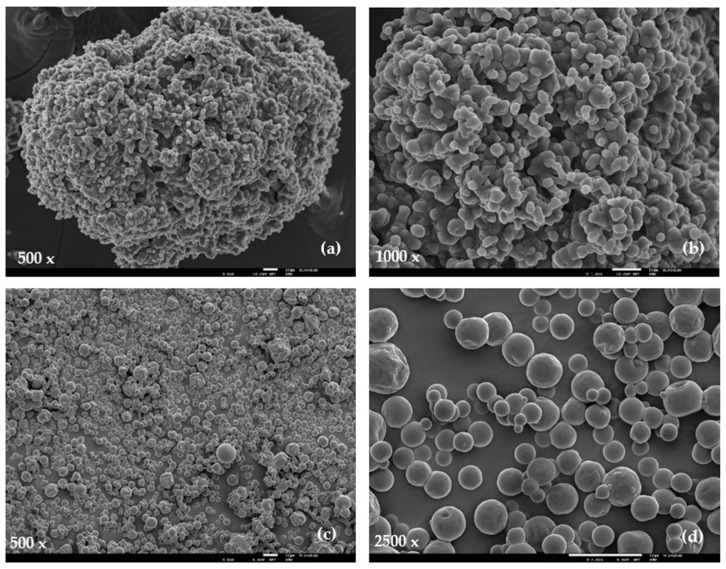
SEM images of particles with encapsulated extracts. Spray dried water extract after being freeze dried (SD-W/FD) (**a**,**b**); spray dried ethanol-water (80:20) extract (SD-E:W 80:20) (**c**,**d**).

The SEM images of particles with encapsulated extracts show different morphologies, depending on the extraction solvents and stabilization methods (Figure 3).

The powder produced by spray drying, upon encapsulation of the freeze dried water extract (SD-W/FD), using maltodextrin as wall material, presents a high degree of microparticles agglomeration, with smaller spherical particles located on the surface of larger ones, with strong interactions between each other (Figure 3a,b). The particles within the agglomerates show mean diameters ranging from 2.9 to 6.3 µm. This agglomerated structure may provide additional stability to the encapsulated extract due to the protection that outer particles may confer to the inner ones. No surface imperfections (e.g., cracks or collapses) are observed. The absence of these imperfections may envisage a good protection and retention of the extract molecules [84]. These agglomerations of particles was also referred to in other works, such as for powders obtained by spray drying orange peel and pulp extracts with a whey protein isolate as an encapsulating agent at 150 °C [85], powder of pomegranate juice with maltodextrin produced at 140 °C [86], and pineapple juice powders with 20% of maltodextrin [87]. According to these authors, the particles produced have this structure as a result of the particles’ high hygroscopicity that starts to establish liquid bridges to connect each other using the available moisture or absorbing the moisture from the environment, and therefore their morphology changes very quickly. Moreover, Tonon et al. [88] revealed that the degree of polymerization (dextrose equivalent) of maltodextrin and the chemical structure of each encapsulated agent influenced the hygroscopicity of powder. Particles produced with the maltodextrin lower polymerization degree showed a lower moisture adsorption rate.

The powder obtained by spray drying the SD-E:W 80:20 extract after the addition of maltodextrin as a wall material has shown more regular and spherical particles, with smooth surfaces and without agglomerates (Figure 3c,d). Lower size particles were compared to the powder obtained with the SD-W/FD extract. The diameter of particles varied between 0.8 and 10.4 µm, however, more than 50% of the particles have a diameter lower than 4 µm. A similar behavior was verified for the ethanol extract of cactus pear powders encapsulated by spray drying with maltodextrin as a wall material [89].

Since the wall material and drying conditions used were the same when encapsulating both SD-W/FD and SD-E:W 80:20 extracts, the different powder properties in terms of particles size and agglomeration may be explained by the fact that ethanol may have not been completely removed from the SD-E:W 80:20 extract prior to encapsulation, influencing the drying process. In addition, the extracts are expected to have different chemical compositions, also influencing differently the hygroscopicity of the obtained particles and consequently the degree of agglomeration.

#### 3.2.3. Phenolic Profile before and after Encapsulation

**Figure 4:**
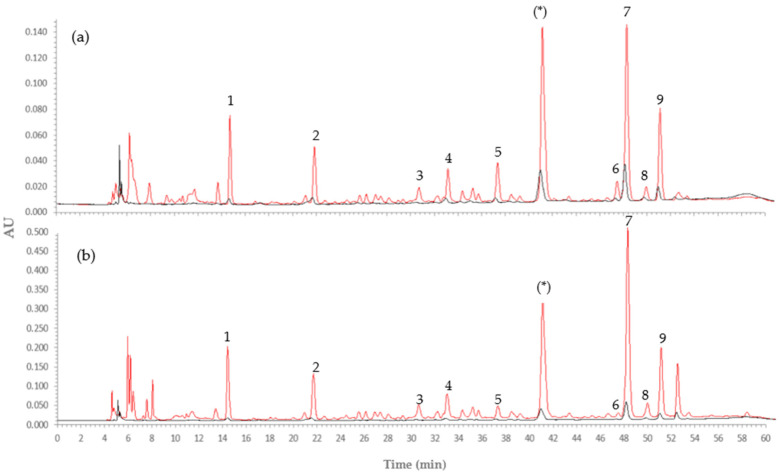
HPLC chromatograms of phenolic compounds detected. (**a**) In water extract W/FD (red line) and in particles with encapsulated water extract W/FD (black line); (**b**) in E:W 80:20 extract (red line) and in particles with encapsulated E:W 80:20 extract (black line). 1. Gallic acid; 2. Hydroxytyrosol; 3. Chlorogenic acid; 4. Dihydroxicaffeic acid; 5. Caffeic acid; 6. P-coumaric; 7. Ferulic acid; 8. Myricetin; 9. Isoferulic acid; (*) unidentified compound.

Some of the phenolic compounds present in the W/FD and E:W 80:20 extracts, before and after encapsulation, were detected and quantified by HPLC. The results are presented in Figure 4 and Table 6. Figure 4 shows the chromatograms of both extracts, in which nine compounds were identified as gallic acid, hydroxytyrosol, chlorogenic acid, dihydroxicaffeic acid, caffeic acid, *p*-coumaric acid, ferulic acid, myricetin, and isoferulic acid, by comparing their retention time with that of standard compounds. These phenolics are some of the major phenolics reported in the literature for pineapple pulp and peel [24,74,90]. One of the main peaks observed (*) was not identified according to the used standards library. However, in the literature, sinapyl derivates peaks were reported as the major compounds in pineapple juice concentrate, including sinapyl-L-cysteine, N-Υ-L- glutamyl-s-sinapyl-L-cysteine, and s-sinapyl glutathione [91]. Moreover, Steingass et al. [92] reported that those phenolics are exclusive of the pineapple fruit and derived foodstuff, which may indicate that this peak can be associated with these compounds.

The most representative peaks of the W/FD extract are the same found in the E:W 80:20 extract, with ferulic acid being the peak with the highest intensity in both extracts (Table 6). However, the intensity of all the peaks is higher in the E:W 80:20 extract, consistent to its higher TPC determined previously (Table 4). The results show that the main peaks observed in the extracts are also present when analyzing the compounds present in the particles after encapsulation, though with lower intensity.

#### 3.2.4. LC-HRMS/MS Analysis

A deeper characterization of the E:W 80:20 extract phenolic composition was performed by liquid chromatography coupled to high resolution tandem mass spectrometry (LC-HRMS/MS). In order to identify the main peak observed by HPLC (*) and not identified by the standards library, a preliminary analysis by LC-DAD-MS was performed. Results are presented as a Supplementary Materials (Figure S1 and Table S1). Forty-five compounds were tentatively identified and displayed a polyphenolic profile similar to that reported in previous studies of pineapple peel [92,93,94,95,96,97]. A peak assignment was based on accurate *m*/*z* values released as deprotonated molecules [M-H]^−^, taking into account the accuracy and precision of the measurement parameters, such as error and Sigma. Each molecular formula was validated by extracting the ion chromatograms from the raw data, and the accurate mass, isotopic and fragmentation pattern were evaluated, and confirmed by literature data.

The HR tandem mass spectrometric data, indicates that the unidentified peak in the E:W 80:20 extract (Figure 4b) is due to an hydroxycinnamoyl (HCA) conjugate acid, the deprotonated molecule of the caffeoyl isocitric acid with *m*/*z* 353.0514 (Table S1, peak 36). The more abundant polyphenol identified on the extract is also an HCA isocitric acid, the p-coumaroyl-isocitric acid displayed a deprotonated molecule with *m*/*z* 337.0565 (Table S1, peak 41). Both molecules are assigned based on the two MS diagnostic peaks observed in the MS tandem ESI negative spectra, the fragment ions with *m*/*z* 154.9986 and 111.0105 (Table S1). According to Masike et al. [97], HCAs conjugated to quinic acid and HCAs conjugated to isocitric acid display different fragmentation paths under the CID analysis. Although both QA and IA precursor ions dissociate yielding intense fragments with *m*/*z* 191 and 173, only the IA with *m*/*z* 191 is prone to rearrange leading to the above assigned MS diagnostic ions. The same molecules were also identified by Steingass et al. [92] and Campos et al. [44] in pineapple extracts. According to these authors, the accurate mass analysis suggested that most compounds, such as caffeic acid, ferulic, synapic acid, and derivatives are HCAS glycosides.

From all the results obtained regarding extraction and stabilization processes studied in this work, further studies were carried out regarding the encapsulation of the E:W 80:20 extract with other wall materials beyond maltodextrin, and the application of the encapsulates on beef meat preservation [38,98].

## 4. Conclusions

Phenolic compounds of pineapple peel were extracted, and their stabilization were explored for further application. From the solid-liquid extraction conditions tested, the one delivering an extract with a higher total phenolic content and antioxidant capacity was a single extraction step with the solvent:pineapple peel ratio of 1:1 (*w*/*w*) for 25 min at ambient temperature, using ethanol-water (80–20%) as a solvent. The direct addition of wall material (maltodextrin) to this extract, followed by its encapsulation by spray drying, was shown as a method with a great potential for the stabilization of its phenolic compounds. Enriched microparticles with a good antioxidant activity compared to the initial extract were produced, possessing a potential positive effect on health beyond basic nutrition. The production of this bioactive powder challenges the fruit sector to move to a circular economy, bringing important advances in terms of the decrease of the environmental impact of the pineapple peel, using low cost extraction processes followed by immediate extracts’ stabilization procedures for future applications. These applications may include new functional food products, products with increased shelf life or active packaging films.

## Figures and Tables

**Table 1:** Levels of the independent variables and total phenolic content (TPC) values. Abbreviations: t—time; S:S—solvent:pineapple peel ratio; PP—pineapple peel; GAE—gallic acid equivalents.

			Independent Variable		Response Variable	
Run	X_1_	X_2_	t (min)	S:S (ratio)	TPC	
					mg GAE/g dry PP	mg GAE/L
1	−1	−1	11	1.6	1.7	134.8
2	−1	1	11	4.4	4.4	122.4
3	1	−1	39	1.6	2.8	219.2
4	1	1	39	4.4	4.6	128.8
5 (C)	0	0	25	3.0	3.4	142.7
6 (C)	0	0	25	3.0	3.4	145.6
7	−α	0	5	3.0	3.1	132.9
8	α	0	45	3.0	3.9	166.1
9	0	−α	25	1.0	2.1	257.9
10	0	α	25	5.0	4.4	101.9
11 (C)	0	0	25	3.0	3.3	138.8
12 (C)	0	0	25	3.0	3.4	146.3

**Table 2:** Adjusted equation from RSM. Abbreviations: S:S—solvent:pineapple peel ratio; t—time.

Dependent Variable TPC	Equation	R^2^	R^2^_adj_	Lack of Fit
mg GAE/g dry PP	3.36 * + 0.60 t * + 0.15 t^2^ + 1.95 S:S * − 0.112 S:S^2^ − 0.42 tS:S	0.97	0.95	0.034
mg GAE/L	143.18 * + 34.20 t * − 0.13 t^2^ − 80.57 S:S * + 29.64 S:S^2^ + 29.64 tS:S	0.89	0.80	0.003

* Significant at the 0.05 level.

**Table 3:** Major physicochemical properties of water extract (W) and ethanol 80% extract (E:W 80:20) produced at ambient temperature.

Assay		Results ^1^	
		W	E:W 80:20
	Moisture content (%)	95.47 ^a^	93.72 ^b^
	pH	4.02 ± 0.02 ^a^	3.70 ± 0.04 ^b^
Color	L*	89.86 ± 0.71 ^b^	92.01 ± 0.20 ^a^
	a*	0.58 ± 0.08 ^a^	−1.55 ± 0.15 ^b^
	b*	−1.01 ± 0.34 ^b^	4.03 ± 0.17 ^a^
TSS	°Brix	5.01 ± 0.05 ^b^	7.80 ± 1.40 ^a^
	wt%	4.50 ± 0.02 ^b^	6.40 ± 0.04 ^a^
TPC	Direct (mg GAE/g dry extract)	2.71 ± 0.059 ^b^	11.10 ± 0.01 ^a^
	Folin Ciocalteau (mg GAE/g dry extract)	4.08 ± 0.46 ^b^	7.05 ± 0.01 ^a^
AOA	DPPH (µmol Trolox/g dry extract)	7.28 ± 2.10 ^b^	91.79 ± 1.98 ^a^
	FRAP (µmol Trolox/g dry extract)	51.19 ± 2.93 ^b^	174.50 ± 9.98 ^a^
	FRAP (µmol Ferrous sulfate/g dry extract)	82.97 ± 4.88 ^b^	285.86 ± 6.64 ^a^

^1^ Means ± standard deviation (*n* = 3 replicates). Different lower-case letters in the same line show statistically significant differences among values (*p* < 0.05).

**Table 4:** TPC of extracts before and after processing by freeze and spray drying.

	TPC	
	Direct Method	Folin
	mg GAE/g dry extract	mg GAE/g dry extract
W	2.71 ± 0.06 ^b^	4.08 ± 0.46 ^a^
E:W 80:20	11.14 ± 0.01 ^b^	7.05 ± 0.01 ^a^
	mg GAE /g freeze dried	
FD-W	7.18 ± 0.02 ^A^	
	mg GAE/g particles	
SD-W/FD	6.12 ± 0.01 ^B^	
SD-E:W 80:20	2.74 ± 0.01 ^C^	

Means ± standard deviation (*n* = 3 replicates). Abbreviations: W—water extract; E:W 80:20—ethanol-water extract; FD-W—freeze dried water extract; SD-W/FD—spray dried water extract after being freeze dried; SD-E:W 80:20—spray dried ethanol-water (80:20) extract. Different letters in the same column show statistically significant differences among values (*p* < 0.05). Lower-case and upper-case letters correspond to extracts and freeze/spray dried particles, respectively.

**Table 5:** AOA of extracts before and after processing by freeze and spray drying.

		W	E:W 80:20	FD-W	SD-W/FD	SD-E:W 80:20
DPPH	TEAC (µmol Trolox/mg GAE)	2.66 ± 0.77 ^c^	8.23 ± 0.18 ^b^	1.59 ± 0.13 ^d^	2.61 ± 0.15 ^c^	10.49 ± 0.09 ^a^
FRAP	TEAC (µmol Trolox/mg GAE)	18.88 ± 1.08 ^a^	15.66 ± 0.90 ^b^	8.33 ± 0.39 ^c^	7.46 ± 0.09 ^c^	15.66 ± 0.27 ^b^
	µmol Sulfate ferrous/mg GAE	30.62 ± 1.80 ^a^	25.65 ± 1.50 ^b^	13.66 ± 0.66 ^c^	12.17 ± 0.15 ^c^	25.52 ± 0.46 ^b^

Means ± standard deviation (*n* = 3 replicates). Abbreviations: W—water extract; E:W 80:20—ethanol-water extract; FD-W—freeze dried water extract; SD-W/FD—spray dried water extract after being freeze dried; SD-E:W 80:20—spray dried ethanol-water (80:20) extract. Different letters in the same line show statistically significant differences among values (*p* < 0.05).

**Table 6:** Quantification of phenolic compounds in the extracts of pineapple peel (mg/100 g dry matter (DM)) and in particles from spray drying (mg/g particles).

Phenolic Compounds	Chemical Formula	Water Extract (W/FD)	E:W 80:20 Extract	Encapsulated W/FD Extract	Encapsulated E:W 80:20 Extract
		mg/100 g DM	mg/100 g DM	mg/g Particles	mg/g Particles
Gallic acid	C_7_H_6_O_5_	27.54	61.13	34.73	18.85
Chlorogenic acid	C_16_H_18_O_9_	16.60	58.04	18.85	20.80
Caffeic acid	C_9_H_8_O_4_	22.41	19.60	14.98	11.13
*p*-Coumaric acid	C_9_H_8_O_3_	18.71	13.00	24.95	21.77
Ferulic acid	C_10_H_10_O_4_	80.23	259.16	89.90	113.92
Total by HPLC		165.49	410.93	183.41	186.47

## Supplementary Materials

The following are available online at https://www.mdpi.com/article/10.3390/foods10061255/s1. Figure S1: UHPLC-HRMS/MS total extract ion chromatogram acquired in the ESI negative mode of an E:W 80:20 extract of pineapple peel. For peak assignment, see Table S1; Table S1: HPLC-ESI-HRMS/MS identification of main polyphenolic compounds in the E:W 80:20 extract of pineapple peel.
