# Supplementary material for: Optimization of Natural Antioxidants Extraction from Pineapple Peel and Their Stabilization by Spray Drying
Source: Foods. 2021 Jun 1;10(6):1255. doi: 10.3390/foods10061255 (PMC8228717; doi:10.3390/foods10061255)
Supplement: Supplementary file 1 [file foods-10-01255-s001.zip › New file SM-Figure S1 and Table S1 (3).pdf]

Optimization of natural antioxidants extraction from pineapple peel and their stabilization by spray drying

Sofia C. Lourenço [1], Débora A. Campos [2], Ricardo Gómez-García [2], Manuela Pintado [2], M. Conceição Oliveira [3], Diana I. Santos [1], Luiz C. Corrêa-Filho [1], Margarida Moldão-Martins [1], and Vítor D. Alves [1,*]

1 LEAF, Linking Landscape, Environment, Agriculture and Food, Instituto Superior de Agronomia, Universidade de Lisboa, Tapada da Ajuda, 1349-017 Lisbon, Portugal; sofiaclourenco@isa.ulisboa.pt, dianaisasantos@isa.ulisboa.pt, lucaalbernaz@gmail.com, mmoldao@isa.ulisboa.pt

2 Universidade Católica Portuguesa, CBQF - Centro de Biotecnologia e Química Fina – Laboratório Associado, Escola Superior de Biotecnologia, Rua Diogo Botelho 1327, 4169-005 Porto, Portugal; deborancampos@gmail.com, rgarcia@porto.ucp.pt, mpintado@porto.ucp.pt

3 Centro de Química Estrutural, Instituto Superior Técnico, Universidade de Lisboa, Av. Rovisco Pais, 1049-001 Lisboa, Portugal; conceicao.oliveira@tecnico.ulisboa.pt

* Correspondence: vitoralves@isa.ulisboa.pt; Tel.: +351-21-365-3546

Supplementary Material

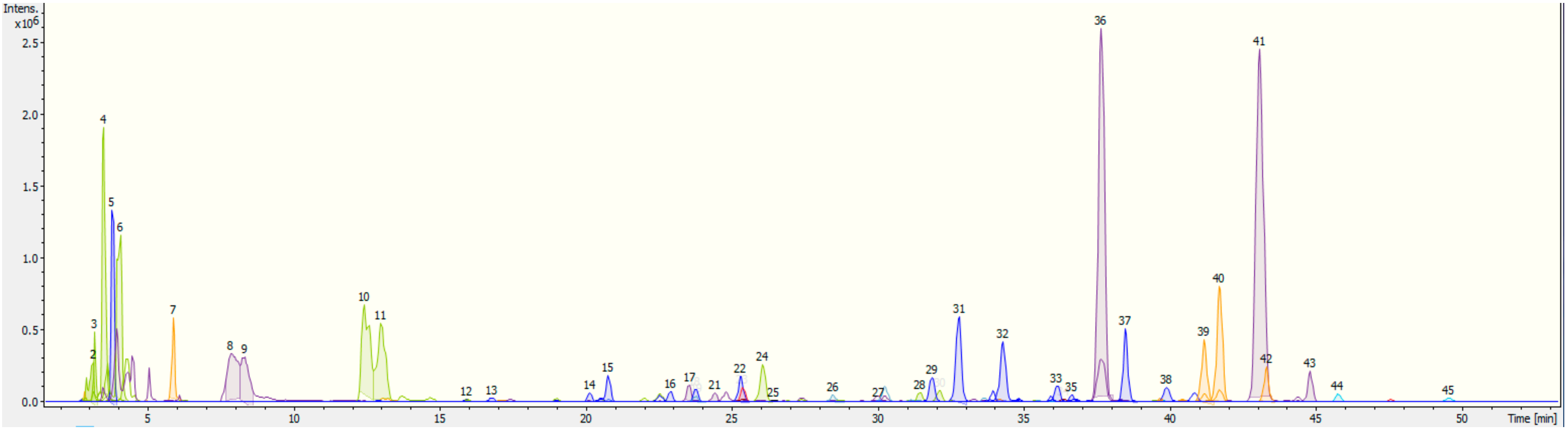

Figure S1. UHPLC-HRMS/MS total extract ion chromatogram acquired in the ESI negative mode of an E:W 80:20 extract of pineapple peel. For peak assignment, see Table S1.

Table S1. HPLC-ESI-HRMS/MS identification of main polyphenolic compounds in E:W 80:20 extract of pineapple peel.

Peak	t_R min	λ max	Proposed structure	[M-H]$^-$ Calc. *m/z*	Meas. *m/z*	Δ (ppm)	MS^2 [(*m/z*) (Δ ppm) (attribution) (%)]	Proposed compound
**1**	2.87	n.d	$C_{10}H_{16}N_2O_7$	275.0885	275.0890	(-1.9)	213.0862 (-7.9) [$C_9H_{13}N_2O_4$]- (40)<br>128.0369 (-8.8) [$C_5H_6NO_3$]- (100)	Glutamyl-glutamic acid
**2**	3.16	n.d	$C_6H_{12}O_6$	179.0561	179.0560	(0.4)	161.0454 (0.8) [$C_6H_9O_5$]- (40)<br>113.0239 (4.8)[$C_5H_5O_3$]- (100)	Galactose
**3**	3.20	n.d	$C_{12}H_{20}O_{12}$	355.0882	355.0887	(-1.5)	293.0886 (-2.7) [$C_{11}H_{17}O_9$]- (15)<br>161.0450 (3.5) [$C_6H_9O_5$]- (60)<br>179.0563 (-1,1) [$C_6H_{11}O_6$]- (100)	3-O-β-D-Galactopyranuronosyl-D-galactose
**4**	3.53	n.d	$C_6H_{10}O_8$	209.0303	209.0307	(-2.1)	191.0197 (-0.7) [$C_6H_7O_7$]- (40)<br>115.0019(12.1) [$C_4H_3O_4$]- (100)	Sacharic acid or isomers
**5**	3.81	n.d	$C_7H_{12}O_6$	191.0561	191.0560	(0.7)	127.0414 (9.3) [$C_6H_7O_3$]- (100)	Quinic acid
**6**	4.09	n.d	$C_{12}H_{22}O_{11}$	341.1089	341.1090	(-0.3)	179.0557 (2.1) [$C_6H_{11}O_6$]- (100)	Sucrose or isomers
**7**	5.90	n.d	$C_{10}H_{17}N_3O_6S$	306.0765	306.0772	(-2.1)	128.0369 (-10.3) [$C_5H_6NO_3$]-(100)	D-d-Glutamyl-L-cysteinglycine
**8**	7.86	n.d	$C_6H_8O_7$	191.0197	191.0195	(-1.0)	173.0094 (-1.2) [$C_6H_5O_6$]- (20)<br>111.0097 (-8.2) [$C_5H_3O_3$]- (100)	Citric acid

Peak	t_R min	λ max	Proposed structure	[M-H]⁻ Calc. *m/z*	Meas. *m/z*	Δ (ppm)	MS[2] [(*m/z*) (Δ ppm) (attribution) (%)]	Proposed compound
**9**	8.35	n.d	$C_6H_8O_7$	191.0197	191.0198	(-0.5)	173.0096 (-2.2) $[C_6H_5O_6]^-$ (100)	Isocitric acid
**10**	12.44	n.d	$C_{18}H_{30}O_{15}$	485.1512	485.1512	(-0.0)	425.1297 (0.8) $[C_{16}H_{25}O_{13}]^-$ (20)<br>365.1096 (-1.7) $[C_{14}H_{21}O_{11}]^-$ (40)<br>221.0668 (-0.6) $[C_8H_{13}O_7]^-$ (100)<br>161.0455 (0.1) $[C_6H_9O_5]^-$ (40)	a-D-Glucopyranuronosyl-(1→2)-6-deoxy-a-L-galactopyranosyl-(1->3)-deoxy-L-galactopyranose
**11**	13.01	n.d	$C_{18}H_{30}O_{15}$	485.1512	485.1515	(-0.6)	221.0669 (-0.6) $[C_8H_{13}O_7]^-$ (100)<br>179.0562 (-0.6) $[C_6H_{16}O_6]^-$ (40)<br>161.0455 (0.1) $[C_6H_9O_5]^-$ (40)	a-D-Glucopyranuronosyl-(1→2)-6-deoxy-a-L-galactopyranosyl-(1->3)-deoxy-L-galactopyranose isomer
**12**	15.96	n.d	$C_{15}H_{16}O_{11}$	371.0620	371.0625	(-1.4)	209.0302 (0.5) $[C_6H_9O_8]^-$ (100)<br>191.0194 (1.9) $[C_6H_7O_7]^-$ (60)	Caffeoylglucaric acid
**13**	16.82	n.d	$C_{14}H_{18}O_9$	329.0878	329.0884	(-1.6)	167.0355 (1.1) $[C_8H_7O_4]^-$ (100)	Vanilloyl-hexoside
**14**	20.18	n..d	$C_{14}H_{18}O_9$	329.0878	329.0886	(-2.6)	167.0352 (1.1) $[C_8H_7O_4]^-$ (70)<br>152.0112 (1.9) $(C_7H_4O_4]^-$ (100)	Vanilloyl-hexoside isomer

Peak	t_R min	λ max	Proposed structure	[M-H]- Calc. *m/z*	Meas. *m/z*	Δ (ppm)	MS[2] [(*m/z*) (Δ ppm) (attribution) (%)]	Proposed compound
**15**	20.80	n.d	$C_{14}H_{20}O_8$	315.1085	315.1090	(-1.3)	135.0434 (12.0) $[C_8H_7O_2]^-$ (100)	(3,4-Dihydroxyphenyl)ethyl D-glucopyranoside
**16**	22.92	n.d	$C_{14}H_{18}O_9$	329.0878	329.0886	(-2.6)	167.0355 (1.1) $[C_8H_7O_4]^-$ (70)<br>123.0452 (8.9) $[C_7H_7O_2]^-$ (100)	Vanilloyl-hexoside isomer
**17**	23.57	n.d	$C_8H_{12}O_7$	219.0510	219.0515	(-2.3)	111.0092 (-4.3) $[C_5H_3O_3]^-$ (100)	Dimethyl citrate
**18**	23.78	n.d	$C_{15}H_{20}O_{10}$	359.0984	359.0988	(-1.3)	197.0456 (-0.5) $[C_9H_9O_5]^-$ (100)	Glucosyringic acid
**19**	23.82	n.d	$C_{16}H_{18}O_{11}$	385.0776	385.0779	(-0.7)	193.0490 (8.5) $[C_{10}H_9O_4]^-$ (80)<br>134.0365 (6.5) $[C_8H_6O_2]^-$ (100)	O-Feruloylaldarate
**20**	24.10	n.d	$C_{16}H_{28}O_{11}$	395.1559	395.1562	(-0.7)	251.1148 (-4.6) $[C_{10}H_{19}O_7]^-$ (100)<br>161.0458 (-1.7) $[C_6H_9O_5]^-$ (50)	1-(3-Methylbutanoyl)-6-Apiosylglucose
**21**	24.47	n.d	$C_{15}H_{18}O_9$	341.0878	341.0877	(0.3)	221.0460 (-2.0) $[C_{11}H_9O_5]^-$ (40)<br>161.0247 (-1.5) $[C_9H_5O_3]^-$ (100)	Caffeoyl glucose
**22**	25.32	n.d	$C_{15}H_{20}O_{10}$	359.0984	359.0988	(-1.1)	239.0564 (-1.5) $[C_{11}H_{11}O_6]^-$ (80)<br>197.0452 (1.6) $[C_9H_9O_5]^-$ (100)	Glucosyringic acid isomer

Peak	t_R min	λ max	Proposed structure	[M-H]⁻ Calc. *m/z*	Meas. *m/z*	Δ (ppm)	MS² [(*m/z*) (Δ ppm) (attribution) (%)]	Proposed compound
**23**	25.40	n.d	$C_{14}H_{20}O_7$	299.1136	299.1143	(-2.1)	119.0516 (12.1) $[C_8H_7O]^-$ (100)	2-(3-hydroxyphenyl)ethanol 1′-glucoside
**24**	26.00	n.d	$C_{14}H_{19}NO_{10}$	360.0936	360.0937	(-0.1)	249.0625 (-3.8) $[C_9H_{13}O_8]^-$ (100)<br>110.0253 (-12.2)$[C_5H_4NO_2]^-$ (50)	1-(Pyrrole-2-carboxyl)-glucuronosylglycerol
**25**	26.46	n.d	$C_{13}H_{16}O_{10}$	331.0671	331.0671	(-0.1)	168.0060 (2.7) $[C_7H_4O_5]^-$ (100)<br>125.0256 (-7.6) $[C_6H_5O_3]^-$ (60)	Galloylglucose
**26**	28.50	n.d	$C_{16}H_{18}O_{11}$	385.0776	385.0778	(-0.5)	193.0519 (-6.6) $[C_{10}H_9O_4]^-$ (80)<br>134.0364 (7.1) $[C_8H_6O_2]^-$ (100)	O-Feruloylgalactarate
**27**	30.06	n.d	$C_{16}H_{18}O_9$	353.0878	353.0875	(1.0)	191.0564 (-1.3) $[C_7H_{11}O_6]^-$ (100)	Caffeoylquinic acid
**28**	31.46	n.d	$C_{14}H_{20}O_9$	331.1035	331.1036	(-0.4)	167.0341 (5.0) $[C_8H_7O_4]^-$ (40)<br>127.0415 (-10.1) $[C_6H_7O_3]^-$ (100)	(3,4,5-Trihydroxyphenyl)ethyl -glucopyranoside
**29**	31.88	n.d	$C_{16}H_{20}O_9$	355.1035	355.1032	(0.7)	175.0399 (1.1) $[C_{10}H_7O_3]^-$ (80)<br>160.0164 (1.0) $[C_9H_4O_3]^{-\cdot}$ (100)	Feruloylglucose

Peak	t_R min	λ max	Proposed structure	[M-H]$^-$ Calc. *m/z*	Meas. *m/z*	Δ (ppm)	MS^2 [(*m/z*) (Δ ppm) (attribution) (%)]	Proposed compound
**30**	31.46	d.d	$C_{14}H_{20}O_9$	331.1035	331.1036	(-0.4)	179.0351 (-0.5) [$C_9H_7O_4$]- (20)<br>127.0415 (-10.1) [$C_6H_7O_3$]- (100)	(3,4,5-Trihydroxyphenyl)ethyl-glucopyranoside isomer
**31**	32.82	328	$C_{17}H_{22}O_{10}$	385.1140	385.1147	(-1.7)	205.0506 (0.3) [$C_{11}H_9O_4$]- (70)<br>190.0270 (0.8) [$C_{10}H_6O_4$]- (100)<br>175.0033 (2.0) [$C_9H_3O_4$]- (20)	Sinapoylglucose
**32**	34.32	n.d	$C_{12}H_{14}O_6$	253.0718	253.0724	(-2.4)	161.0243 (0.9) [$C_9H_5O_3$]- (100)<br>133.0283 (8.7) [$C_8H_5O_2$]- (90)	Caffeoyl glycerol
**33**	36.16	n.d	$C_{24}H_{38}O_{12}$	517.2291	517.2286	(-0.9)	385.1863 (1.2) [$C_{19}H_{29}O_8$]- (20)<br>293.0887 (-2.9) [$C_{11}H_{17}O_9$]- (50)<br>205.1234 (-0.1) [$C_{13}H_{17}O_2$]- (100)	Vomifoliol 9-[xylosyl-(1->6)-glucoside]
**34**	36.41	n.d	$C_{16}H_{18}O_8$	337.0942	337.0927	(0.7)	163.0401 (5.8) [$C_9H_7O_3$]- (100)	Coumaroylquinic acid
**35**	36.61	212; 318	$C_{27}H_{35}N_3O_6$	496.2353	496.2449	(0.4)	346.1774 (-0.4) [$C_{18}H_{24}N_3O_4$]- (100)<br>149.0598 (-2.1) [$C_7H_7N_3O$]- (20)	N1,N10-Diferuloylspermidine

Peak	t_R min	λ max	Proposed structure	[M-H]$^-$ Calc. *m/z*	Meas. *m/z*	Δ (ppm)	MS^2 [(*m/z*) (Δ ppm) (attribution) (%)]	Proposed compound
**36**	37.68	214; 328	$C_{15}H_{14}O_{10}$	353.0514	353.0518	(-1.0)	191.0196 (0.7) [$C_6H_7O_7$]- (90)<br>173.0090 (1.1) [$C_6H_5O_6$]- (100)<br>154.9986 (-2.9 [$C_6H_3O_5$]- (20)<br>111.0105 (-11.8) [$C_5H_3O_3$]- (10)	Caffeoyl-isocitric acid
**37**	38.50	n.d	$C_{27}H_{35}N_3O_6$	496.2453	496.2449	(0.8)	346.1776 (-1.2) [$C_{18}H_{24}N_3O_4$]- (100)<br>331.1534 (1.0) [$C_{17}H_{21}N_3O_4$]- (80)	N1,N10-Diferuloylspermidine isomer
**38**	39.94	220; 306	$C_{16}H_{20}O_{10}$	371.0984	371.0982	(0.3)	249.0613 (1.2) [$C_9H_{13}O_8$]- (100)<br>217.0364 (10.2) [$C_8H_9O_7$]- (50)	Dihydroferulic acid 4-O-glucuronide
**39**	41.20	215; 268	$C_{20}H_{27}N_3O_8S$	468.1426	468.1437	(-2.5)	306.0772 (-2.2) [$C_{10}H_{16}N_3O_6S$]$^-$ (80)<br>272.0895 (-2.4) [$C_{10}H_{14}N_3O_6$]$^-$ (100)<br>254.0790 (-2.9) [$C_{10}H_{12}N_3O_5$]$^-$ (85)<br>210.0889 (-2.2) [$C_9H_{12}N_3O_3$]$^-$ (90)<br>179.0464 (-1.2) [$C_8H_7N_2O_3$]$^-$ (40)	Coniferylglutathione

Peak	t_R min	λ max	Proposed structure	[M-H]⁻ Calc. *m/z*	Meas. *m/z*	Δ (ppm)	MS² [(*m/z*) (Δ ppm) (attribution) (%)]	Proposed compound
**40**	41.72	214; 278	$C_{21}H_{29}N_3O_9S$	498.1531	498.1547	(-3.2)	306.0767 (-0.7) $[C_{10}H_{16}N_3O_6S]^-$ (80)<br>272.0892 (-1.5) $[C_{10}H_{14}N_3O_6]^-$ (100)<br>254.0788 (-2.1) $[C_{10}H_{12}N_3O_5]^-$ (85)<br>210.0886 (-0.8) $[C_9H_{12}N_3O_3]^-$ (90)<br>179.0460 (1.4) $[C_8H_7N_2O_3]^-$ (40)	S-sinapylglutathione
**41**	43.10	210: 314	$C_{15}H_{14}O_9$	337.0565	337.0564	(0.3)	173.0092 (-0.4) $[C_6H_5O_6]^-$ (40)<br>163.0402 (-0.5) $[C_9H_7O_3]^-$ (30)<br>154.9988 (-1.5 $[C_6H_3O_6]^-$ (60)<br>111.0105 (-11.8) $[C_5H_3O_3]^-$(100)	p-Coumaroyl-isocitric acid
**42**	43.34	n.d	$C_{19}H_{26}N_2O_8S$	441.1337	441.1329	(1.9)	249.0556 (-2.2) $[C_8H_{13}N_2O_5S]^-$ (20)<br>225.0595 (-2.0) $[C_{11}H_{13}O_3S)^-$ (90)<br>171.0775 (-0.1) $[C_7N_{11}N_2O_3]^-$ (100)<br>128.0382 (-8.9) $(C_5H_6NO_3]^-$ (60)	N-L-d-Glutamyl-S-sinapyl-L-cysteina
**43**	44.84	215; 328	$C_{16}H_{16}O_{10}$	367.0671	367.0665	(1.6)	173.0094 (-1.2) $[C_6H_5O_6]^-$ (40)<br>154.9990 (-2.9 $[C_6H_3O_6]^-$ (70)<br>111.0105 (-11.8) $[C_5H_3O_3]^-$ (100)	p-Feruloyl-isocitric acid

Peak	t_R min	λ max	Proposed structure	[M-H]$^-$ Calc. *m/z*	Meas. *m/z*	Δ (ppm)	MS^2 [(*m/z*) (Δ ppm) (attribution) (%)]	Proposed compound
**44**	45.78	n.d	$C_{27}H_{30}O_{17}$	625.1410	625.1405	(0.9)	463.0876 (1.4) $[Y_1]^-$ $[C_{21}H_{19}O_{12}]^-$ (100)<br>301.0352 (0.6) $[Y_0]^-$$[C_{15}H_9O_7]^-$ (90)<br>300.0274 (0.4) Y_0-H$]^{-\cdot}$$(C_{15}H_8O_7]^{-\cdot}$ (40)	Quercetin di-O-hexoside
**45**	49.57	n.d	$C_{21}H_{20}O_{12}$	463.0882	463.0877	(1.0)	300.0282 (-2.1) $[Y_0$-H$]^{-\cdot}$ $(C_{15}H_8O_7]^{-\cdot}$ (100)<br>271.0249 (-0.2) $[C_{14}H_7O_6]^-$ (20)	Quercetin
